# Supplementary material for: Validating organoid-derived human intestinal monolayers for personalized therapy in cystic fibrosis
Source: Life Sci Alliance. 2023 Apr 4;6(6):e202201857. doi: 10.26508/lsa.202201857 (PMC10079552; doi:10.26508/lsa.202201857)
Supplement: Supplementary file 1 [file LSA-2022-01857_TableS1.docx]

| Patient | CFTR Mutation | Age at the time of biopsy | Sex | Baseline Sweat Chloride mmol/L | Pancreatic Status | CFTR modulator therapy |
| --- | --- | --- | --- | --- | --- | --- |
| 1 | W1282X/W1282X | 38 | female | 107 | PI | none |
| 2 | W1282X/W1282X | 14 | male | 97 | PI | none |
| 3 | G542X/G542X | 5 | female | 130 | PI | none |
| 4 | G542X/G542X | 17 | male | n/a | PI | none |
| 5 | N1303K/N1303K | 20 | male | n/a | PI | none |
| 6 | N1303K/N1303K | 7 | male | 111 | PI | none |
| 7 | F508del/G85E | 10 | male | n/a | PI | none |
| 8 | F508del/F508del | 12 | male | 90 | PI | ORKAMBI® |
| 9 | F508del/F508del | 38 | female | n/a | PI | ORKAMBI® |
| 10 | F508del/3272-26A*→*G | 59 | female | 114 | PI | none |
| 11 | G551D/F508del | 36 | female | 121 | PI | KALYDECO® |
| 12 | G551D/3272-26A*→*G | 53 | female | 121 | PS | KALYDECO® |
| 13 | F508del/G178R | 24 | female | 102 | PS | KALYDECO® |
| 14 | G551D/5T | 43 | male | 81 | PS | KALYDECO® |

Table S1. CF patient cohort demographics and clinical characteristics.

PI, pancreatic insufficient; PS, pancreatic sufficient
